# Antibacterial Activity and Cytotoxicity of Silver(I) Complexes of Pyridine and (Benz)Imidazole Derivatives. X-ray Crystal Structure of [Ag(2,6-di(CH_2_OH)py)_2_]NO_3_

**DOI:** 10.3390/molecules21020087

**Published:** 2016-01-28

**Authors:** Urszula Kalinowska-Lis, Aleksandra Felczak, Lilianna Chęcińska, Ilona Szabłowska-Gadomska, Emila Patyna, Maciej Małecki, Katarzyna Lisowska, Justyn Ochocki

**Affiliations:** 1 Department of Bioinorganic Chemistry, Medical University of Lodz, Muszyńskiego 1, 90-151 Łódź, Poland; justyn.ochocki@umed.lodz.pl; 2 Department of Industrial Microbiology and Biotechnology, Faculty of Biology and Environmental Protection, University of Lodz, 12/16 Banacha Street, 90-237 Łódź, Poland; afelczak@toya.net.pl (A.F.); emcia.pat@gmail.com (E.P.); katalis@biol.uni.lodz.pl (K.L.); 3 Department of Theoretical and Structural Chemistry, University of Lodz, Pomorska 163/165, 90-236 Łódź, Poland; lilach@uni.lodz.pl; 4 Department of Applied Pharmacy and Bioengineering, Medical University of Warsaw, Banacha 1, 02-097 Warsaw, Poland; igadomska@wum.edu.pl (I.S.-G.); maciej.malecki@wum.edu.pl (M.M.)

**Keywords:** silver(I) complexes, cytotoxicity, B16 melanoma cells, 10T1/2 fibroblasts, antibacterial activity, Hirshfeld surface

## Abstract

Selected aspects of the biological activity of a series of six nitrate silver(I) complexes with pyridine and (benz)imidazole derivatives were investigated. The present study evaluated the antibacterial activities of the complexes against three Gram-negative strains: *Pseudomonas aeruginosa* ATCC 15442, *Escherichia coli* ATCC 25922 and *Proteus hauseri* ATCC 13315. The results were compared with those of silver nitrate, a silver sulfadiazine drug and appropriate ligands. The most significant antibacterial properties were exerted by silver(I) complexes containing benzimidazole derivatives. The cytotoxic activity of the complexes was examined against B16 (murine melanoma) and 10T1/2 (murine fibroblasts) cells. All of the tested silver(I) compounds were not toxic to fibroblast cells in concentration inhibited cancer cell (B16) viability by 50%, which ranged between 2.44–28.65 µM. The molecular and crystal structure of silver(I) complex of 2,6-di(hydroxymethyl)pyridine was determined by single-crystal X-ray diffraction analysis. The most important features of the crystal packing and intermolecular non-covalent interactions in the Ag(I) complex were quantified via Hirshfeld surface analysis.

## 1. Introduction

Due to the rapidly increasing phenomenon of drug resistance, the search for new and effective antimicrobial agents is a worldwide concern. Benzimidazole and pyridine derivatives are known to possess desirable antibacterial, antifungal, antiviral and antiproliferative properties [1,2,3,4,5,6]. Silver compounds are also well known for their antimicrobial properties and are widely used in the treatment of wounds and burn infections [7,8,9,10]. The combination of benzimidazole or pyridine derivatives with silver ions leads the production of compounds with novel biological properties which can be an attractive alternative to existing treatments [11,12,13,14,15].

Many silver compounds have been shown to have antimicrobial activity. Silver sulfadiazine (SSD or AgSD) is the most popular silver drug used in wound therapy and medicinal devices [8,16,17]. Although silver sulfadiazine has been used for many years, the drug causes important adverse effects, including blood dyscrasias, gastrointestinal reactions and allergic reactions [18,19]. Moreover, AgSD delays wound healing due to its cytotoxicity towards fibroblasts and keratinocytes [20,21,22].

Although silver complexes also demonstrate significant antitumor activity, the body of research in this area is still very poor compared with that devoted to other metals’ complexes. Nevertheless, a lot of silver(I) complexes have been found to exhibit greater cytotoxic activity than that of cisplatin, a drug used in cancer treatment, with relatively low toxicity to normal human cells. Ag(I) complexes show selectivity toward various types of tumor cells, and this is dependent on the type of ligand linked to the silver(I) ions. This dependency is probably connected with the stability of the complexes and the hydrophilicity-lipophilicity of the complexes formed by the type of the ligand [23,24,25,26,27].

The present study describes the antibacterial activity and cytotoxic activity of a series of silver(I) complexes (Table 1) [13,28,29]. Phosphate and hydroxymethylene derivatives of pyridine, imidazole, and benzimidazole were chosen for the study. Derivatives of heterocyclic compounds show many pharmacological properties: e.g., some pyridine derivatives (pyridine-3-carboxylic (nicotinic) acid and its amide) play crucial roles in physiological functions, especially as part of the NAD/NADH coenzyme. Importantly, nicotinic acid also acts as an anti-pellagra agent, ensuring good skin and mucosa condition [30,31]. Another selected ligand is metronidazole, a well-known antibiotic drug used to treat certain parasitic and bacterial infections [32].

**Table 1:** Empirical formulae of silver(I) complexes and structural formulae of ligands.

Ag(I) Complex Formula (No.)	Ligand Structure	Ref.
{[Ag(4-pmOpe)]NO_3_}*_n_* (**1**)	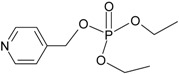 4-pmOpe	[28]
[Ag(2-bimOpe)_2_]NO_3_ (**2**)	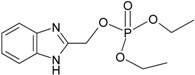 2-bimOpe	[28]
[Ag(2,6-di(CH_2_OH)py)_2_]NO_3_ (**3**)	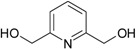 2,6-di(CH_2_OH)py	[29]
[Ag(4-CH_2_OHpy)_2_]NO_3_ (**4**)	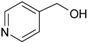 4-CH_2_OHpy	[29]
[Ag(2-CH_2_OHbim)_2_]NO_3_ (**5**)	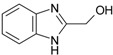 2-CH_2_OHbim	[29]
[Ag(MTZ)_2_NO_3_] (**6**)	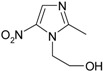 MTZ (metronidazole)	[13]

The antibacterial activity of the tested compounds was determined against three Gram-negative pathogens: *Pseudomonas aeruginosa* ATCC 15442, *Escherichia coli* ATCC 25922, and *Proteus hauseri* ATCC 13315. These microorganisms can cause urinary tract infections, diarrhea, pneumonia, and other life-threatening diseases. The bacteria from this group are also responsible for diverse nosocomial infections [33,34,35].

In addition, as the complexes are intended for direct application to the skin, it uses *in vitro* tests conducted on normal (fibroblasts) and diseased (murine malignant melanoma) model cell lines derived from murine skin to determine the cytotoxicity of the silver(I) complexes.

The study also presents the molecular and crystal structures of the silver(I) complex of 2,6-di(hydroxymethyl)pyridine.

## 2. Results and Discussion

### 2.1. Crystal and Molecular Structure of ***3***

**Figure 1:**
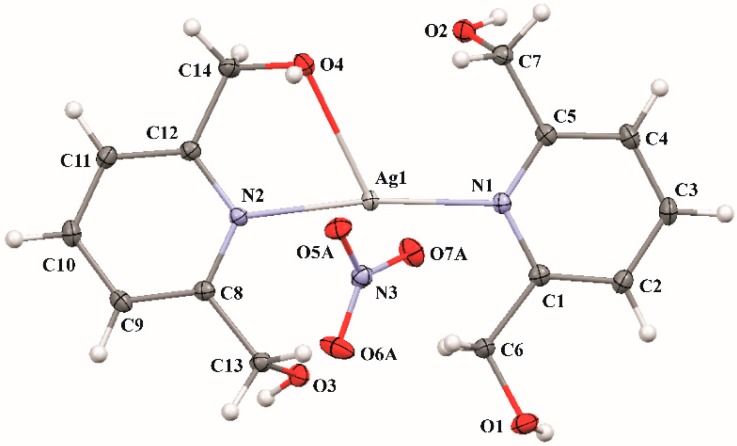
The molecular structure of the title Ag(I) complex (*MERCURY* [36] representation), with atom labels and 50% probability displacement ellipsoids for non-H atoms. Only the major component (A) of disordered oxygen atoms in the anion molecule (NO_3_^−^) is shown (minor components B are omitted for clarity).

**Figure 2:**
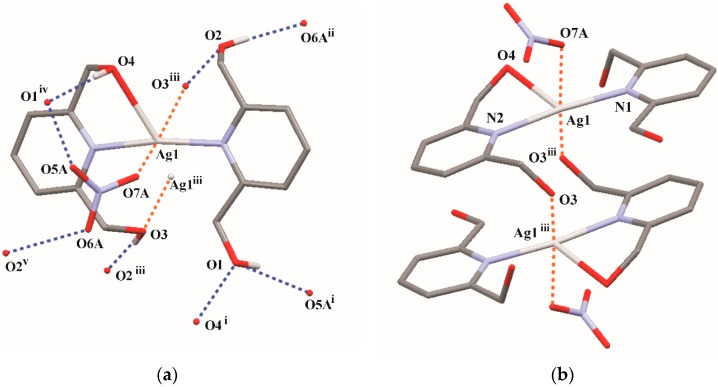
A scheme of the different-type interactions observed in crystal structure of **3** (**a**) and a formation of the centrosymmetric dimer (**b**). Bond distances to Ag1: Ag1–N1 = 2.187(1) Å, Ag1–N1 = 2.198(1) Å, Ag1–O4 = 2.612(1) Å. Intermolecular short contacts to Ag1 (drawn as orange dotted lines): Ag1–O7A^i^ = 2.87(1) Å and Ag1–O3^iii^ = 2.960(1) Å; bond angles: N1–Ag1–N2 = 171.66(4)°, N2–Ag1–O4 = 71.06(4)°. Blue dotted lines indicate intermolecular interactions (for details see Table 3). H-atoms have been omitted for clarity (except of those involved in H-bonds in Figure 2a). Symmetry codes: (i) −*x*, *y* – 1/2, −*z*; (ii) *x* + 1, *y*, *z*; (iii) −*x*, 1 − *y*, −*z*; (iv) −*x*, *y* + ½, −*z* + 1/2; (v) *x* − 1, *y*, *z*.

X-ray crystallography analysis reveals that **3** crystallizes in monoclinic *P*2_1_/c space group. Basic information pertaining to crystal parameters and structure refinement is summarized in Table 2. As illustrated in Figure 1 [36], the asymmetric unit of **3** contains one Ag(I) complex cation and one nitrate anion, both located in general positions. The Ag1 is coordinated by two 2,6-di(CH_2_OH)py (2,6-di(hydroxymethyl)-pyridine) ligands: one of them acts as a monodendate donor through the pyridine nitrogen (N1) atom, in contrary to second one, which behaves as bidendate and interacts with metal centre via the pyridine nitrogen (N2) atom and hydroxyl oxygen (O4) atom. Additionally, one can consider two quite close contacts to Ag1: Ag1–O7A (from nitrate counter anion) as well as Ag1–O3 (−*x*, 1 − *y*, −*z*; from an adjacent complex molecule), both complement the coordination sphere around the silver(I) cation. Thus, the coordination number of Ag1 is 5 (Figure 2a,b). The dihedral angle between the two pyridine best planes is 6.18(7)°, while the N1–Ag1–N2 is 171.66(4)°. The bond distances and angles for 2,6-di(CH_2_OH)py ligands in **3** are within the expected ranges when compared with the structural results presented for free ligand [2,6-di(CH_2_OH)py] [37].

**Table 2:** Crystallographic data for **3**.

	Complex 3
Empirical formula	C_14_H_18_AgN_3_O_7_
Formula weight	448.18
Crystal system	Monoclinic
Space group	*P*2_1_/c
*a* (Å)	9.7304(1)
*b* (Å)	14.7348(2)
*c* (Å)	14.0158(2)
α (°)	90.000
β (°)	127.803(1)
γ (°)	90.000
*V* (Å^3^)	1587.77(4)
*Z*	4
*T* (K)	100(2)
*F*(000)	904
*D_x_* (g·cm^−3^)	1.875
μ (mm^−1^)	1.32
Scan method	ω scan
θ range (°)	2.3–28.0
Measured reflections	27856
Unique reflections	3836
Observed reflections [*I* > 2σ(*I*)]	3729
Completeness to θ_max_ (%)	100
No. of parameters/restraints	271/18
*R* [*I* > 2σ(*I*)]	0.0147
*wR* (all data)	0.0379
*S*	1.09
Largest diff. peak (e·Å^−3^)	0.48
Largest diff. hole (e·Å^−3^)	−0.30

A systematic search of the Cambridge Structural Database [38] indicates that 2,6-di(CH_2_OH)py ligand tends to be tridentate donor in transition metal [Co(II), Ni(II), Cu(II), Zn(II)) complexes [39,40,41], albeit in only two single–crystal structures of Ag(I) complexes, reported in literature, the two hydroxyl groups of the 2,6-di(CH_2_OH)py ligand remain uncoordinated [*refcode* XAKWOW [42]], either only one CH_2_OH arm is engaged in coordinative interaction with the silver ion (*refcode* LIWWIY [43]). In the former structure Ag[(sac)(2,6-di(CH_2_OH)py)] (where sac = saccharinate) structure the relatively short Ag–N distance of 2.1490(15) Å was found. In the latter structure, the silver-donor bond lengths and related angle [Ag–O=2.614(3) Å and Ag–N=2.180(3) Å, N–Ag–O=71.57(10)°] correspond well with those determined for **3** (see caption of Figure 2).

By the use of coordinative Ag–O contacts, two adjacent complex cation molecules together with two accompanying nitrate anions form a dimeric unit lying across centre of symmetry. Within such dimers the pyridine rings are π-π stacked with a Cg1···Cg2^iii^/Cg2···Cg1^iii^ distance of 3.914(1) Å and corresponding interplanar distances of 3.489(1) Å/3.597(1) Å; *Cg*1 and Cg2 are the centroids of the N1/C1–C5 and N2/C8–C12 rings, respectively (Figure 2b).

Furthermore, all four hydrogen groups act as proton donors in intermolecular O–H···O hydrogen bonds. The geometries of interactions, presented in Figure 2a, are summarized in Table 3. Finally, in the crystal structure of the Ag(I) complex, **3**, interacting molecules built a 3D hydrogen-bonded network.

**Table 3:** Hydrogen bonding geometry (Å, °) for **3**.

	D-H	H···A	D···A	D-H···A
O1-H1A···O5A^i^	0.81(2)	1.94(2)	2.709(5)	159(2)
O2-H2A···O6A^ii^	0.78(2)	1.95(2)	2.721(5)	170(2)
O3-H3A···O2A^iii^	0.81(2)	1.89(2)	2.691(2)	169(2)
O4-H4A···O1A^iv^	0.82(2)	1.94(2)	2.726(2)	163(2)

Symmetry codes: (i) −*x*, *y* − 1/2, −*z*; (ii) *x* + 1, *y*, *z*; (iii) −*x*, 1 − *y*, −*z*; (iv) −*x*, *y* + 1/2, −*z* + 1/2.

To provide further insight into the packing and intermolecular contacts in the analyzed structure of complex **3**, the Hirshfeld surface analysis [44] was performed using CrystalExplorer [45] software. The Hirshfeld surface is defined by the outer contour of the space which a molecule of interest consumes in a crystalline environment [46]. A ratio *d*_norm_ (given by Equation (1)) encompassing the distances of any surface point to the nearest atom outside (*d_e_*) and inside (*d_i_*) the surface and the van der Waals radii of the atoms ($r_{i}^{vdW}$ and $r_{e}^{vdW}$, respectively) enables the identification of the regions with individual intermolecular interactions unique for the crystal structure [47,48]:

(1) $$d_{\mathrm{norm}}\;=\;\frac{d_{i}\;-\;r_{i}^{vdW}}{r_{i}^{vdW}}\;+\;\frac{d_{e}\;-\;r_{e}^{vdW}}{r_{e}^{vdW}}$$

A red–white–blue color scheme is characteristic for the Hirshfeld surface projection with *d*_norm_ function mapped onto it: red color highlights short contacts, white color is used for weak contacts around the van der Waals separation, and blue color corresponds for a region to be free of significant contacts.

The associated two-dimensional histograms being a combination of *d_i_ vs. d_e_*, referred to as “fingerprint plots”, are useful tool in identifying and comparing different kinds of intermolecular interactions. Fingerprint plots are unique for a given crystal structure [49].

**Figure 3:**
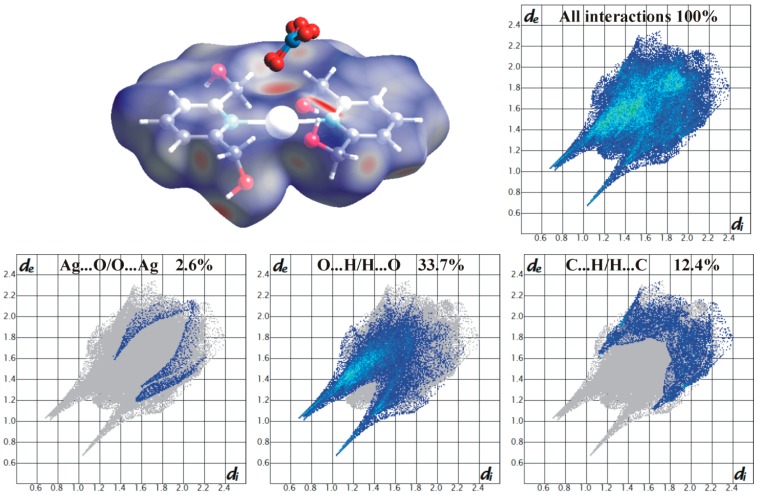
Hirshfeld surface of Ag(I) complex-cation molecule with the geometrical function *d*_norm_ mapped onto it (a colour scale from red to blue: −0.75 Å–1.2 Å) and corresponding finger plots indicating all types of contacts and these reduced to contact types as following Ag···O/O···Ag, O···H/H···O, C···H/H···C [the distances from a surface point to the nearest interior/exterior atoms (*d_i/e_*) are given in Å].

Figure 3 presents the Hirshfeld surface modelled over the cation molecule, [Ag(2,6-di(CH_2_OH)py)_2_]^+^ and corresponding two-dimensional fingerprint plots showing all types of contacts and these reduced to selected contact types: Ag···O/O···Ag, O···H/H···O, C···H/H···C.

As expected, the H···H contacts comprise 39% of the surface area due to the small van der Waals radius of hydrogen and packing effects in molecular structures [50], likewise the H···H distances observed in **3** are typical without extremely short ones.

Furthermore, the O···H/H···O contacts play a dominate role with a significant contribution of 33.7%. It was found that CH_2_OH arms of the 2,6-di(CH_2_OH)py ligands are mostly involved in quite strong O-H···O hydrogen bonds (the shortest interactions are shown as long spikes) and weaker C-H···O interactions. Another type of weak C···H/H···C contacts give only 12.4% of the surface. The aromatic π-π stacking interactions mainly represented by C···C and C···N contacts (4.4% and 1.4%, respectively) as well as N···H/H···N contacts (3.5%) make a small contribution to the Hirshfeld surface comparable to the all interactions with metal Ag1 atom (4.2%). Among short contacts of Ag···X (X = O,N,C,H) dominate the Ag···O ones (2.6%).

### 2.2. Antibacterial Activity

**Figure 4:**
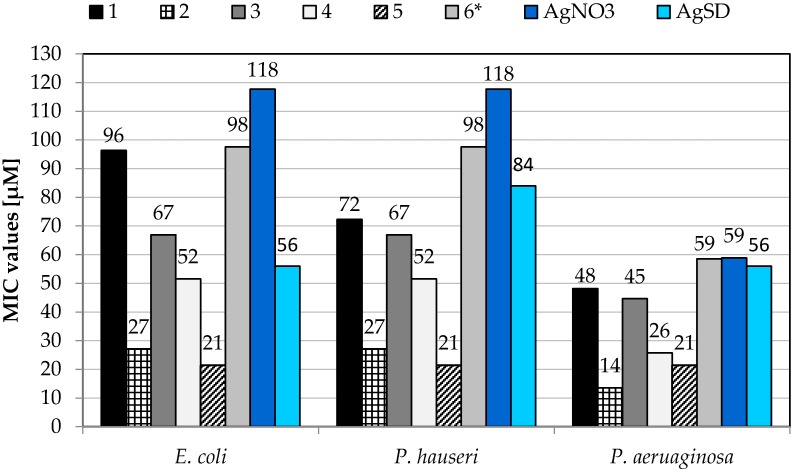
Antibacterial activity of the complexes **1**–**6**, given as MIC (minimum inhibitory concentration) values in µmol/L^−1^. MIC values of the free ligands, *i.e.*, 4-pmOpe, 2-bimOpe, 2,6-di(CH_2_OH)py, 4-CH_2_OHpy, 2-CH_2_OHbim, are >500 mg/L^−1^. AgSD: silver sulfadiazine. The all experiments were performed in triplicate (SD = 0). * Complex **6** [13].

The antibacterial properties were expressed as MIC (minimum inhibitory concentration) and MBC (Minimum Bactericidal Concentration) values. The activity of silver compounds **1**–**6**, were compared with those of two commonly-used silver antimicrobial agents (AgNO_3_ and AgSD) andthe appropriate free ligands (Figure 4). The studies indicate that hydroxymethylene and phosphate derivatives of pyridine and benzimidazole did not exhibit any antibacterial activity up to 500 mg/L^−1^, while all the tested silver complexes possess relatively good antimicrobial properties. Among them, complex **2** exhibited the highest antibacterial activity. It inhibited the growth of *P. aeurignosa* at a concentration almost 4.5-fold lower than that of AgSD. In the case of *E. coli* and *P. hauseri*, the MIC value was 27 µM, which was also much lower than that observed for AgSD. Complexes **1** and **2** were previously characterized as compounds showing good antibacterial and antifungal activity [28]. Compound **1** exhibited antimicrobial activity against *E. coli* ATCC 8739 and *P. aeruginosa* ATCC 27853 with MIC values 37.6 µM for both strains. Also compound **2** inhibited the growth of mentioned bacteria, at concentrations of 21.2 µM for *E. coli* ATCC 8739 and 5.3 µM for *P. aeruginosa* ATCC 27853 [28].

Satisfactory antibacterial properties were also displayed by complex **5**, which inhibited the growth of *E. coli* and *P. aeruginosa* at a concentration 2.6-fold lower than that determined for AgSD. Its MIC value against *P. hauseri* was almost four-fold lower than that of reference AgSD. In the case of *P. aeruginosa*, complex **5** demonstrated good antimicrobial activity and inhibited the growth of bacteria at a concentration almost three-fold lower than that of AgSD. The MBC values (Table 4) obtained for the tested complexes also confirmed that, among the analyzed group, complexes **2** and **5** demonstrated the highest antibacterial activity and were more active than the reference compounds (AgNO_3_ and AgSD). To summarise, complexes **2** and **5** containing a benzimidazole ring in their structure demonstrated much higher activity than that containing pyridine. Moreover, mentioned compounds were more effective than AgSD and AgNO_3_ toward the selected tested Gram-negative pathogens.

**Table 4:** MBC (minimum bactericidal concentration) values (µM) of the tested compounds.

Compound	*E. coli* ATCC 25992	*P. hauseri* ATCC 13315	*P. aeruginosa* ATCC 15442
**1**	145	120	72
**2**	54	41	27
**3**	67	112	67
**4**	77	77	52
**5**	21	43	21
**6** [13]	98	117	117
AgNO_3_	177	177	59
AgSD	84	140	56

Our previous studies also indicated that silver(I) complex **6**, containing metronidazole and nitrate counter-ion, exhibited satisfactory antimicrobial properties against tested Gram-negative bacteria. Antimicrobial activity of **6** was lower or comparable with those presented by AgNO_3_ [13].

Silver *N*-heterocyclic carbene complexes are widely described in the literature as effective antibacterial, antifungal, and antiviral agents [1]. Haque *et al.* synthesized dinuclear silver complexes with *N*-heterocyclic carbene, which possessed good antibacterial activity. The authors also demonstrated that compounds containing benzimidazole moiety exhibited significantly higher activity than complexes with imidazole [51]. The 4-vinylbenzyl-substituted benzimidazol-2-ylidene silver complex effectively inhibited the growth of Gram-positive and Gram-negative bacteria and demonstrated good antifungal activity against yeast [52]. Moreover, newly synthesized silver complexes can constitute an excellent alternative for the treatment of infections caused by antibiotic-resistant bacteria. Silver compounds containing 4,5,6,7-tetrachlorobenzimidazole showed high antibacterial activity against methicillin-resistant *Staphylococcus aureus* strains or silver-resistant *Escherichia coli* [53].

### 2.3. Cytotoxic Activity

**Figure 5:**
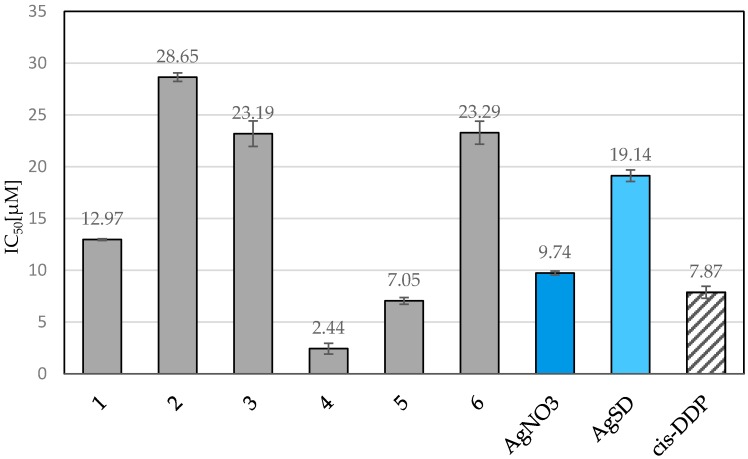
Response of B16 cell lines to silver complexes **1**–**6**, AgNO_3_, AgSD (silver sulfadiazine) and *cis*-DDP (cisplatin). Values presented are IC_50_ (µM) ± SD for three independent experiments.

Firstly, the *in vitro* cytotoxic activity of silver complexes **1**–**6** and free ligands towards murine melanoma (B16) cell lines was determined and compared with that of AgNO_3_, AgSD (silver sulfadiazine) and *cis*-DDP (cisplatin). The IC_50_ values (µM), *i.e.*, the concentrations of compounds that cause 50% reduction of cellular viability, are given in Figure 5. The percentage of viable B16 cells treated with the silver(I) complexes and referenced compounds is given in Figure S1. The results show meanvalue ± SD of three repetitions.

The IC_50_ values of complexes **1**–**6** range from 244 to 2865 µM. Complexes **4** and **5** demonstrate the highest cytotoxicity against B16 cells, with respective IC_50_ values of 2.44 and 7.05 µM. The activity of complex **4** is four times greater than that of AgNO_3_ and eight times greater than AgSD. Complex **5** demonstrates slightly greater cytotoxicity than AgNO_3_ and about twice as much as AgSD. Moreover, complex **5** exhibits IC_50_ value in the same range as cisplatin, whose IC_50_ value is 7.87 µM, whereas complex **4** is three-fold higher in IC_50_ value compared to cisplatin. Though complexes **4** and **5** contain different heterocyclic rings, *i.e.*, pyridine and benzimidazole, they both include the hydroxymethylene groups, the presence of which may determine the activity of the complexes.

However, the cytotoxic activity of the free ligands is very low (>200 µM), their presence is crucial for activity of the complex as a whole. It is hard to conclude how the ligands influence the cytotoxic activity of the complexes, the barrier is the lack of a defined mechanism of action of Ag(I) complexes. It has been previously proposed that the activity of the silver complexes is not only dependent on the metal ions but is connected with the presence of the metal together with the ligands [54,55].

**Figure 6:**
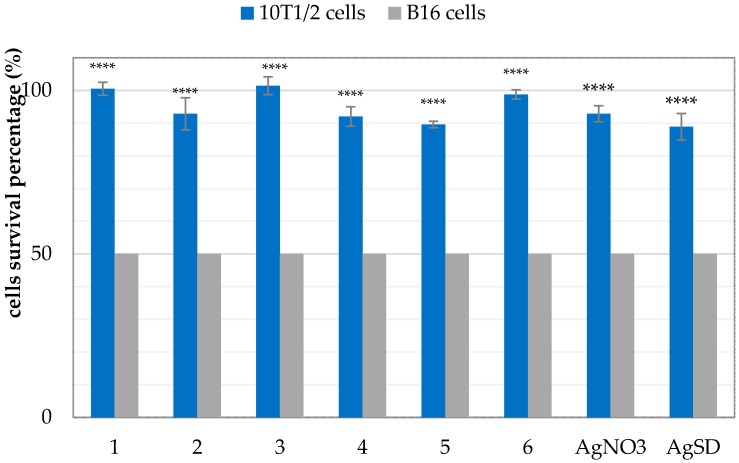
Percentage of survival 10T1/2 cells treated with complexes **1**–**6**, AgNO_3_ and AgSD, in concentrations that correspond with IC_50_ values for B16 cells. Statistical analyses were done using GraphPad Prism 6 and one-way ANOVA with Bonferroni’s multiple comparisons test. *p* < 0.05 was considered as statistically significant one. Data were showed as mean with SD of three independent experiments. **** *p* < 0.0001.

Secondary, in order to evaluate the effect of silver(I) complexes **1**–**6** and referenced silver compounds (AgNO_3_ and AgSD) on non-cancerous cells, we also examined their cytotoxicity on murine fibroblasts cells, 10T1/2. The percentage of survival 10T1/2 cells was measured just for concentrations, in which 50% of murine melanoma cells (B16) remain viable (IC_50_ values). The results (Figure 6) are very promising, because the percentage of survival of non-cancerous cells (10T1/2) ranges from 90% to 100%. This means that all tested silver compounds are not toxic to fibroblast cells in concentration inhibited cancer cell viability by 50%. The tested silver(I) complexes could be potential anticancer agents.

The limited amount of research concerning the cytotoxicity of silver(I) complexes towards mentioned cell lines (B16 and 10T1/2) exists. Cytotoxicity studies of silver(I) complexes of substituted (2-aminophenyl)phosphines have shown them to be active against B16 cells in the range of comparable to cisplatin (IC_50_ 0.9 µM) [56]. Similarly, the other group of silver(I) complexes containing chiral tertiary phosphine ligands has been examined towards B16 cells and their IC_50_ values ranged from 1.3 to 5.0 µM [27].

## 3. Materials and Methods

### 3.1. Synthesis of the Complexes ***1**–**6***

The syntheses of complexes **1**–**6** were performed according to procedures reported previously [13,28,29]. The reference compounds: AgNO_3_ (POCH, Gliwice, Poland), silver sulfadiazine AgSD (Sigma-Aldrich Sp. z.o.o, Poznań, Poland), and cisplatin (Sigma-Aldrich Sp. z.o.o); the ligands: 2,6-di(CH_2_OH)py, 4-CH_2_OHpy, 2-CH_2_OHbim (Sigma-Aldrich Sp. z.o.o), MTZ (Sigma-Aldrich Sp. z.o.o) were all used as supplied. The ligands: 4-pmOpe and 2-bimOpe was synthesized according to previously described procedures [57,58].

### 3.2. X-ray Single-Crystal Structure Determination

The X-ray measurement of **1** was performed on an Agilent SuperNova diffractometer (Agilent Technologies, Yarnton, England) with an Atlas detector using Mo*K*_α_ radiation and ω scan at 100 K. Multi-scan absorption [59] correction was applied to correct the measured X-ray intensities. The structure was solved and refined using SHELXL-2013 [60]. During the refinement the extinction correction was also required. All non-hydrogen atoms were refined anisotropically. H atoms bonded to oxygen atoms were located in a difference map and refined with the O–H distance restrained to a common value of 0.84 Å. Other (C)–H atoms were positioned geometrically and refined using a riding model with C–H = 0.95–0.99 Å and with *U*iso(H) = 1.2 *U*eq(C) or 1.5 *U*eq(methyl C).

Three oxygen atoms (O5, O6, O7) in nitrate group were found to be disordered and refined with the final site-occupation factors for the two alternative positions to *k_A_*:*k_B_* = 0.69(3):0.31(3). Moreover, geometrical similarity constraints were applied to all N–O bonds within the disordered anion molecule, using the SADI instruction.

CCDC1405058 contains the supplementary crystallographic data for this paper. These data can be obtained free of charge via http://www.ccdc.cam.ac.uk/conts/retrieving.html (or from the CCDC, 12 Union Road, Cambridge CB2 1EZ, UK; Fax: +44 1223 336033; E-mail: deposit@ccdc.cam.ac.uk).

### 3.3. Antibacterial Activity Studies

The antimicrobial susceptibility testing of synthesized complexes, free ligands, and references compounds (AgNO_3_ and AgSD) was performed against three Gram-negative strains: *Eschericha coli* ATCC 25922, *Proteus hauseri* ATCC 13315, and *Pseudomanos aeruginosa* ATCC 15442. The minimum inhibitory concentration (MIC) and minimum bactericidal concentration (MBC) were determined by a modified broth microdilution method according to the recommendation of Clinical and Laboratory Standards Institute (CLSI M07-A8). In the experiments the Mueller-Hinton broth was used, the final organism density was about 5 × 105 CFU/mL and the tests were performed 20 h at 37 °C. The studied compounds were dissolved in water (**1**, **3**, **4**, **6,** and the ligands [except 2-CH_2_OHbim]) or in DMSO (**2**, **5,** and 2-CH_2_OHbim), and the appropriate amounts were added to samples (to biotic controls was added pure solvent). The compounds were tested in concentration ranging from 0 mg/L to 100 mg/L, except free ligands which were tested up to 500 mg/L.

### 3.4. Cytotoxic Activity. MTT Assays

The B16 (murine melanoma) cells and C3H10T1/2 (mouse fibroblast) cells [61] were seeded 7 × 10^3^ (B16) and 3 × 10^4^ (10T1/2) per well/24 well plate into a medium containing DMEM GlutaMAX (Thermo Fisher Scientific, Waltham, MA USA), 10% bovine serum (Lonza, San Diego, CA, USA) added, and Antibiotic Antimicotic Solution 1:100 (Sigma-Aldrich). The plates were stored in a humidified incubator at 37 °C, 5% CO_2_. After 24 h, the cells were treated with the following compounds: compounds **1**–**6**, free ligands, AgNO_3_, and AgSD (except control cells—wells without tested compound). Silver complexes **1**, **3**, **4**, **6**, and AgNO_3_ were dissolved in water. Complexes **2**, **5**, and AgSD are DMSO-soluble. Next, all chemicals were diluted into water to make a concentration 1, 0.1, 0.01 mg/mL and as a working solution were added to the medium. After 72 h of incubation, the cells were washed and incubated with 0.25 mg/mL MTT (Sigma-Aldrich) solution and were incubated for another 3 h. Isopropanol was added to dissolve the formazan crystals. Absorbance at 540 and 690 nm was read using Epoch Software version 2.01.14 (Bio-Tek, Winooski, VT, USA).

## 4. Conclusions

Antibacterial and cytotoxic activity of a series of silver(I) complexes of phosphate and hydroxymethylene derivatives of pyridine and (benz)imidazole was determined. Crystal and molecular structure of silver(I) complex with 2,6-di(hydroxymethyl)pyridine (**3**) was completed herein.

The complexes containing benzimidazole derivatives, **2** and **5**, were the most active against Gram-negative strains: *Pseudomonas aeruginosa* ATCC 15442, *Escherichia coli* ATCC 25922, and *Proteus hauseri* ATCC 13315. Their antibacterial potency was higher than that of referenced drug AgSD (silver sulfadiazine) and AgNO_3_. Moreover the complex **2** exhibited antibacterial activity at concentrations that were not toxic to the eukaryotic cells.

The *in vitro* cytotoxicity has been assessed against the cancerous B16 (murine melanoma) and non-cancerous 10T1/2 (murine fibroblasts) cell lines. Two of the tested silver(I) complexes containing hydroxymethylene groups, *i.e.*, **4** and **5**, displayed greater cytotoxic activity against B16 cells (murine melanoma) than that of AgNO_3_, AgSD, and cisplatin. All the complexes **1**–**6** were found to have relatively low toxicity to non-cancerous 10T1/2 cells (murine fibroblasts). The percentage of survival of 10T1/2 cells treated with the mentioned compounds in concentrations equal to IC_50_ values for B16 cells, was in the range of 90%–100%. Results are favourable. Any potential anticancer agents should be non-toxic for healthy cells and toxic towards cancer. 

## Supplementary Materials

Supplementary materials can be accessed at: http://www.mdpi.com/1420-3049/21/2/87/s1.
