# Supplementary material for: Antibacterial Activity and Cytotoxicity of Silver(I) Complexes of Pyridine and (Benz)Imidazole Derivatives. X-ray Crystal Structure of [Ag(2,6-di(CH2OH)py)2]NO3
Source: Molecules. 2016 Jan 28;21(2):87. doi: 10.3390/molecules21020087 (PMC6274122; doi:10.3390/molecules21020087)
Supplement: Supplementary file 1 [file molecules-21-00087-s001.pdf]

Molecules **2016**, 21, 87; doi:10.3390/molecules21020087

Supplementary Materials: Antibacterial Activity and Cytotoxicity of Silver(I) Complexes of Pyridine and (Benz)Imidazole Derivatives. X-ray Crystal Structure of $[Ag(2,6\text{-}di(CH_2OH)py)_2]NO_3$

Urszula Kalinowska-Lis, Aleksandra Felczak, Lilianna Chęcińska, Ilona Szabłowska-Gadomska, Emila Patyna, Maciej Małecki, Katarzyna Lisowska and Justyn Ochocki

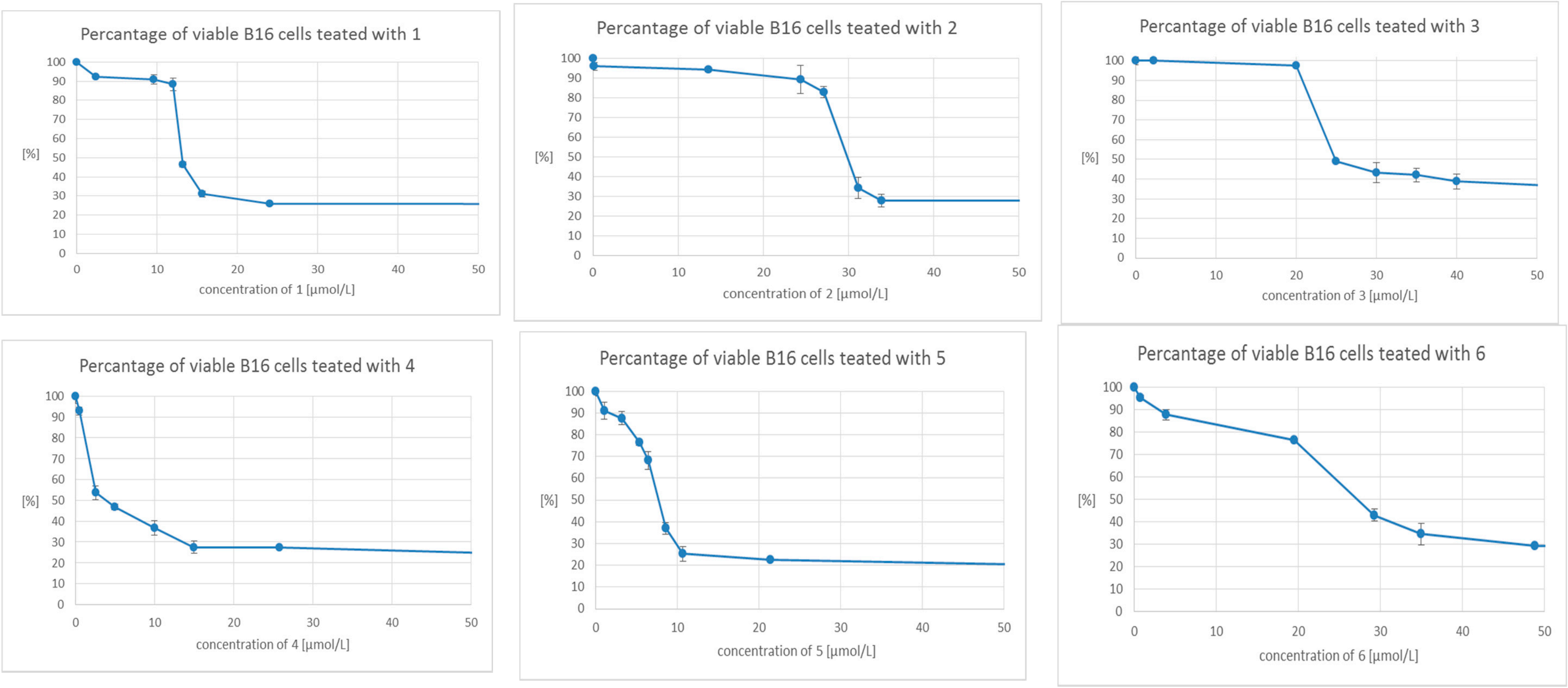

Figure S1. *Cont.*

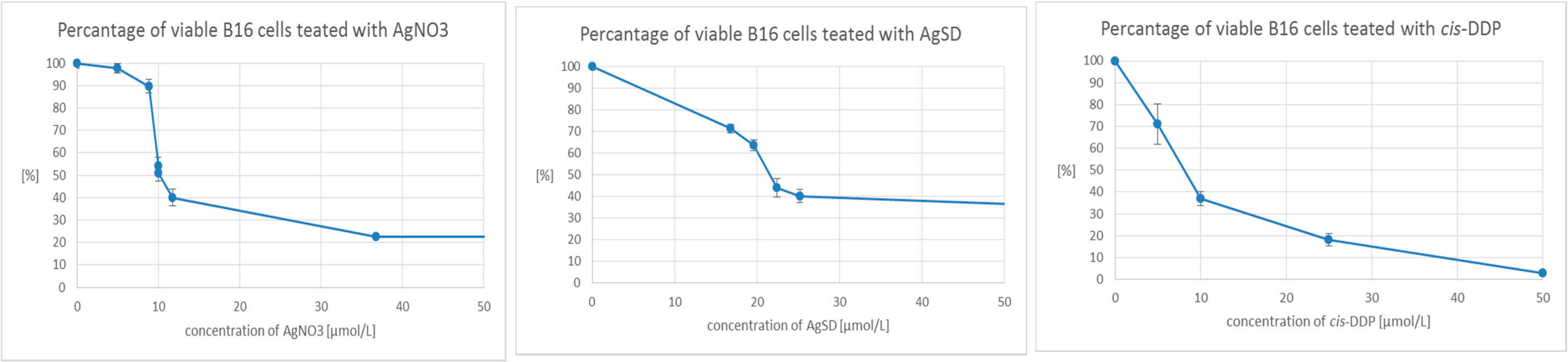

Figure S1. Cytotoxic activity of the silver(I) complexes **1–6** and referenced compounds: $AgNO_3$, AgSD (silver sulfadiazine) and *cis*-DDP (cisplatin) towards B16 murine melanoma cell lines. Results obtained by means of MTT assay and shown as mean value ± SD.
